# Institutionalizing community-focused maternal, newborn, and child health strategies to strengthen health systems: A new framework for the Sustainable Development Goal era

**DOI:** 10.1186/s12992-017-0259-z

**Published:** 2017-06-26

**Authors:** William T. Story, Karen LeBan, Laura C. Altobelli, Bette Gebrian, Jahangir Hossain, Judy Lewis, Melanie Morrow, Jennifer N. Nielsen, Alfonso Rosales, Marcie Rubardt, David Shanklin, Jennifer Weiss

**Affiliations:** 1 The University of Iowa, College of Public Health, Iowa City, IA USA; 2 Independent Consultant (formerly CORE Group), Washington, DC USA; 3 Future Generations University, Franklin, WV USA; 4 University of Connecticut School of Medicine, Farmington, CT USA; 5 CARE Bangladesh, Dhaka, Bangladesh; 6 Maternal and Child Survival Program and ICF, Washington, DC and Rockville, MD USA; 7 Helen Keller International, New York, NY USA; 8 World Vision US, Washington, DC USA; 9 Independent Consultant (formerly CARE USA), Vashon, WA USA; 10 Concern Worldwide US, New York, NY USA

**Keywords:** Health systems, Scale-up, Non-governmental organizations, Maternal, newborn, and child health, Community-focused strategies

## Abstract

**Background:**

Stronger health systems, with an emphasis on community-based primary health care, are required to help accelerate the pace of ending preventable maternal and child deaths as well as contribute to the achievement of the Sustainable Development Goals (SDGs). The success of the SDGs will require unprecedented coordination across sectors, including partnerships between public, private, and non-governmental organizations (NGOs). To date, little attention has been paid to the distinct ways in which NGOs (both international and local) can partner with existing national government health systems to institutionalize community health strategies.

**Discussion:**

In this paper, we propose a new conceptual framework that depicts three primary pathways through which NGOs can contribute to the institutionalization of community-focused maternal, newborn, and child health (MNCH) strategies to strengthen health systems at the district, national or global level. To illustrate the practical application of these three pathways, we present six illustrative cases from multiple NGOs and discuss the primary drivers of institutional change. In the first pathway, “learning for leverage,” NGOs demonstrate the effectiveness of new innovations that can stimulate changes in the health system through adaptation of research into policy and practice. In the second pathway, “thought leadership,” NGOs disseminate lessons learned to public and private partners through training, information sharing and collaborative learning. In the third pathway, “joint venturing,” NGOs work in partnership with the government health system to demonstrate the efficacy of a project and use their collective voice to help guide decision-makers. In addition to these pathways, we present six key drivers that are critical for successful institutionalization: strategic responsiveness to national health priorities, partnership with policymakers and other stakeholders, community ownership and involvement, monitoring and use of data, diversification of financial resources, and longevity of efforts.

**Conclusion:**

With additional research, we propose that this framework can contribute to program planning and policy making of donors, governments, and the NGO community in the institutionalization of community health strategies.

## Background

The health of women, children, and adolescents is a central component of the new Sustainable Development Goals (SDGs). The Global Strategy for Women’s, Children’s, and Adolescents’ Health (2016–2030) lays out a broad strategy for achieving the health-related SDGs, especially the ending of preventable maternal, child, newborn and adolescent deaths with a greater focus on enabling all citizens of the world to achieve their full health potential [1]. Both the SDGs and the Global Strategy call for a significant paradigm shift on the part of national governments, non-governmental organizations (NGOs), development aid organizations, and the private sector. Success will require strategic, action-oriented partnerships; unprecedented coordination across sectors; and a focus on prevention through locally-led approaches [1, 2].

An emerging consensus among global health leaders is that building stronger health delivery systems will require more than multi-sectoral collaboration. An emphasis on community-based primary health care will be critical for the future fight against the top killers of children and mothers around the world [3]. Community-focused strategies such as home visitation, community mobilization, formation of community-based support groups, and task-shifting to community health workers have the maximum potential to improve a range of maternal, newborn, and child health interventions [4, 5]. Newer approaches to global health have been put forth in the Paris Declaration on Aid Effectiveness [6], in reports of the World Health Organization's Commission on Social Determinants of Health [7], and most recently the Post-2015 SDGs, namely a commitment to locally-driven interventions across sectors, a focus on innovation to find new strategies for reaching marginalized groups, and broader advocacy at national and international levels.

Expanding the impact of locally-specific, multi-sectoral, innovative approaches to health will require careful attention to the most appropriate strategies, normative frameworks, financing mechanisms, and political and policy decision-making pathways to achieve scale. Experience suggests that rather than increasing funding and rapidly expanding health service coverage (i.e., horizontal scaling up), donors and implementing organizations (both public and private) should focus on engaging key stakeholders, using data to make decisions, and incorporating results from pilot projects to inform policy and, ultimately, strengthen health systems (i.e., vertical scaling up) [8]. Horizontal scaling up occurs when a program or intervention expands its size by replication in different locations or by increasing the number of beneficiaries in a given location [9–11]. On the other hand, according to Simmons and colleagues [10], “vertical or political scaling up takes place when innovations are institutionalized through policy or legal action. Systems and structures are adapted and resources redistributed to build the institutional mechanisms that can ensure sustainability (p. 13).” In the current paper, our conceptualization of scale aligns with vertical scaling up, namely the institutionalization of effective community-focused health strategies to strengthen existing health systems at the district, national, or global level [9]. Civil society organizations (including international and local NGOs), government organizations, and private bussinesses have the potential to apply this form of scaling up to create an environment for long-term, country-led impact [10, 12].

Currently, there is a dearth of literature on the distinct ways in which NGOs (both international and local) contribute to institutionalizing community-focused health strategies in partnership with national health systems. Previous work on scaling-up has focused on topics such as family planning and reproductive health [10, 13], HIV/AIDS [14], and other development initiatives [15, 16]. Recently, Smith and colleagues [17] examined the rapid expansion of technical interventions in maternal and newborn health led by one organization—Jhpiego. In the current paper, we examine the roles undertaken by a variety of NGOs in the expansion of community-focused maternal, newborn, and child health (MNCH) initiatives, with a focus on a functional shift that goes beyond the expansion of a technical intervention to the institutionalization of MNCH strategies that strengthen existing health systems. We present a conceptual framework that depicts three primary, non-exclusive, and often complementary pathways through which NGOs have facilitated the institutionalization of community-focused approaches to improve MNCH at the district, national and global levels. To illustrate the practical application of these three pathways, we present six examples, or cases, from multiple NGOs. We also discuss the primary drivers of institutional change that were found to be critical for the success of community-focused MNCH strategies.

## Conceptual framework

**Fig. 1:**
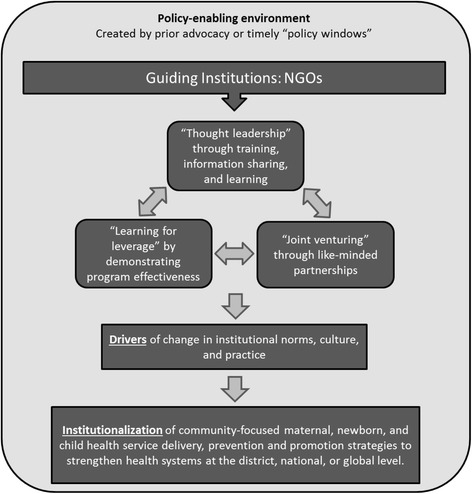
Conceptual framework for the institutionalization of community-focused maternal, newborn and child health strategies into government health systems

The conceptual framework is based on practical experiences of NGOs that have been engaged in community-focused MNCH programs for over 25 years as well as current literature on scale-up, implementation science, health systems strengthening, and evidence-informed policy making. Figure 1 depicts the components of the conceptual framework that facilitate the institutionalization of community-focused MNCH strategies to strengthen existing health systems. The framework starts with an organization, or “guiding institution,” that catalyzes change and provides sustained leadership for community-focused MNCH approaches [17]. The framework specifically focuses on NGOs due to their historical influence on national and global priorities in global health [18]. Health and development NGOs can help advance the maternal and child health targets set forth in the SDGs in countries where health systems may be weak by drawing upon their potential assets, such as their extensive networks, deep local presence within communities, ability to collaborate across sectors, and commitment to working with vulnerable and marginalized populations [19]. Furthermore, some NGOs have strong leaders within each country who are capable of uniting the policy community through existing relationships with Ministry of Health (MOH) and other Ministry officials as well as building local organizational capacity [18]. There are multiple pathways through which NGOs can stimulate change within existing health systems. We present three non-exclusive, complementary pathways that have been shown to be critical to the institutionalization of community-focused MNCH strategies across time and context.

The first pathway, “learning for leverage,” was introduced by Alan Fowler [20] as a strategy for enhancing the effectiveness of NGOs in international development. This pathway refers to the use of innovative community health or service delivery strategies as sources of experimentation and demonstration to inform health system changes [20]. According to Fowler [20], some NGOs have the capacity and experience to reorient government priorities by demonstrating which alternative strategies work. However, simply conducting research to test a new innovation in MNCH is not enough. Organizations seeking to build the evidence for community health strategies must ensure that resources invested in research have an impact on policy and practice by translating results into terms that fit the social and political context [21]. Some NGOs have started to demonstrate the effectiveness and efficiency of community-based approaches to reducing maternal and child morbidity and mortality [22–26]. NGOs can use this evidence to help inform new policies and decisions about resources to support initiatives that can reach more individuals with life-saving interventions at a lower cost, especially those with limited access to health facilities.

The second pathway, “thought leadership,” was presented in the international development literature by Peterson and colleagues [19] and refers to the ability to disseminate lessons learned to multiple audiences to advance better ways of solving MNCH challenges through training, information sharing, and collaborative learning [12, 19]. NGOs can disseminate proven community-focused MNCH interventions by sharing innovative community health strategies with other NGOs [27], other health actors, and national Ministries of Health [28, 29] to improve service delivery and child nutrition. This requires fiscal and operational flexibility to dedicate sufficient resources to develop the capacity of key stakeholders and organizations to address complex MNCH challenges. More specifically, NGOs can develop training and learning sites in communities and districts, especially if they have a long-term presence in these areas.

The third pathway, “joint venturing,” was first described by Uvin and colleagues [12] as a strategy to scale up NGO impact. This pathway refers to working in partnership with other organizations (public or private) to demonstrate the efficacy of a MNCH project and using a collective voice to inform decision-makers [12]. According to Uvin and colleagues [12], the size of the group matters when seeking to affect government policies and programs. It is easier for a large group of NGOs (who have experience managing programs that politicians consider relevant to their own mandate) to motivate key decision-makers to adopt a new program, than for a single NGO (local, national, or international) to have the same effect. Joint venturing not only allows greater coverage of the program, it also increases the visibility of the program for national stakeholders to take interest. However, the benefits of NGO partnerships go beyond the strategies of increasing coverage and visibility through collaboration. According to Levinger and Mulroy [30], the best partnerships accomplish more together than each individual organization can achieve on its own. These consortium efforts create continuity within communities by building on local leadership and trust that NGOs have fostered from different projects, expanding interventions to be more comprehensive and contextually appropriate, and collaborating with other development actors to create economies of scale.

Each of the aforementioned pathways—individually or in combination with one another—has the potential to guide decision-makers at the district, national and global levels so that they actively give attention to issues and strategies that may have not been priorities in the past, and so they can develop stronger and more people-centered health systems. In order to have an impact on institutional norms, specific drivers are needed to move community-focused MNCH strategies forward [11]. These driving factors will help create a cultural shift among government agencies that moves from historically- and bureaucracy-driven mandates to policies that generate community-led accountability in order to achieve success at the grassroots level [11]. Cultural change at both institutional and community levels is needed to empower each side of the government-community partnership for system change [31]. In addition, drivers of change require institutional support and strategic alignment among implementing organizations, policy advocates, and government decision-makers to provide financial, technical, and human resources for community health strategies that address MNCH issues of political importance [17]. Both institutional support and cultural change are important steps toward the institutionalization of community-focused MNCH strategies to strengthen health systems.

Changes in institutional norms and the adaptation of existing health policies do not happen in a vacuum. The policy environment can create many challenges to integrating proven interventions into policy and practice. Policy decisions often depend on the agenda of the government at that moment and not the nature of the evidence [32]. Therefore, it is critical to take advantage of “policy windows,” or opportunities when political priorities align favorably with civil society demand for community-focused strategies that address MNCH issues [17].

## Institutionalization pathways: Six illustrative cases

The following six cases, or examples, illustrate the operationalization of the three pathways discussed above within the context of community-focused MNCH projects. We selected the cases from 82 U.S. Agency for International Development (USAID)-funded Child Survival and Health Grant Program projects from the last 10 years. We selected six cases that provided a heterogeneous mix of organizational size, country of operation, and intervention focus. Although these cases are from international NGOs, their relevance is equally applicable to local NGOs and other implementing organizations [9–12].

The cases selected represent six countries from three regions (Latin America and Caribbean, sub-Saharan Africa, and South Asia) and six NGOs ranging in size from small to large. Each organization has demonstrated aspects of all three pathways; however, each case focuses on only one of the three pathways. Table 1 displays a summary of the key components of each case. Additional elements of the conceptual framework are also described in each case, including the importance of the policy environment and the drivers of institutional change, which will be discussed after all of the cases are presented. These cases reflect the context in each country at a moment in time, which represents one part of a longer development process.

**Table 1:** Summary of illustrative cases

Country	Organization	Pathway	Description
Bangladesh	CARE	Learning for leverage	CARE was able to demonstrate an increase in service utilization and a decline in maternal mortality by establishing community support groups to identify and track all pregnant women. The Bangladesh government adopted this community health service delivery model at the national level.
Haiti	Haitian Health Foundation	Learning for leverage	HHF provided evidence for the effectiveness of community-based treatment of childhood pneumonia, which was used to inform treatment protocols developed by the Ministry of Population and Public Health, as well as train organizations in the treatment on pneumonia.
Nepal	Helen Keller International	Thought leadership	HKI promoted a nutrition-friendly agricultural strategy (Action Against Malnutrition through Agriculture) to reduce malnutrition in women and young children by bringing together stakeholders from multiple sectors to make collaborative plans at the national and local levels.
Peru	Future Generations	Thought leadership	Future Generations helped incorporate the community in co-management of primary health care services through Local Health Administration Communities (or CLAS). This model was incorporated into national health policy and observational training centers were developed to scale-up the strategy to other areas.
Rwanda	Consortium led by Concern Worldwide	Joint venturing	Building on the results of a home-based management of malaria pilot program by adding pneumonia and diarrhea treatment, this consortium of three NGOs was able to roll-out integrated community case management for all three diseases in six districts by 2008 and made significant contributions towards national scale-up by 2010.
Senegal	Consortium led by ChildFund International	Joint venturing	Over the past 15 years this consortium of six NGOs revitalized the “health huts” initiative and helped it grow into a national program designed to reach rural and urban communities by working in synergy across geographic areas to increase service coverage and improve standards.

## Learning for leverage

### CARE (Bangladesh)

The Community Support System (CmSS) model for addressing maternal mortality has been implemented by CARE in Bangladesh since 1999. Its evolution covered three phases: (1) initial development of the approach with documentation; (2) replication of the approach with additional emphasis on documentation and program modifications, such as increased involvement of local government officials; and (3) greater recognition by both local and national MOH, as well as other stakeholders. The project focused on removing socioeconomic barriers to maternal health service utilization through devising local solutions, which coincided with government initiatives to upgrade and strengthen maternal health services at district and sub-district levels. An essential component of the CmSS model was the establishment of community committees to take responsibility for pregnant women in their village.

The first phase of the intervention began in one sub-district of Dinajpur district and focused on the development of a data system to track service utilization and mortality among pregnant women. The data collected through this project demonstrated an increase in service utilization and decrease in maternal deaths, which led to the second phase of the project [33]. The second phase replicated the Dinajpur intervention package in two sub-districts of Narshingdi, and refined the intervention by involving health center staff and local government officials in project planning and implementation. In 2010, an impact study revealed that CmSS was associated with increased use (and reduction in wealth disparities) of critical maternal health services, including antenatal care, facility delivery and emergency obstetric care. Government officials joined CARE to review and reflect on these project achievements, which enabled CARE to share their positive experiences with CmSS among a wider audience. Using evidence from the second phase of the project, CARE was able to engage national stakeholders through dissemination workshops and donor visits, which garnered attention from the MOH and led to the incorporation of the CmSS model into national health and nutrition plans.

In response to a request from the MOH, the third phase of the CmSS approach began in 2011. During this phase, CARE advised the MOH on how to replicate the CmSS model in 12,584 community clinic catchment areas across 64 districts throughout the country. A variety of donors and 11 national and international NGOs have contributed to the implementation of CmSS through the national Community-Based Health Care (CBHC) program, including United Nations Children’s Fund (UNICEF), Japanese International Cooperation Agency (JICA), Canadian International Development Agency, Bangladesh Rural Advancement Committee, and Plan International.

Several contextual factors also contributed to the successful institutionalization of the CmSS model in Bangladesh. In particular, the active involvement of government officials, health workers, and community members in the collection of high quality, community-level data effectively documented a successful and feasible model for addressing the problem of maternal mortality. Currently, under the CBHC program, the Government of Bangladesh plans to establish three CmSS committees for each of the 13,500 community clinics (covering a total 81 million people).

### Haitian Health Foundation (Haiti)

The Haitian Health Foundation (HHF) was established in 1986 to address community health in the rural Grand′Anse Department of Haiti. It started with a community-oriented primary care focus on child health in the department capital of Jérémie, where pneumonia was, and continues to be, the major cause of child death. In 1990, the World Health Organization (WHO) supported an ethnographic assessment of rural mothers to understand their explanatory models for the symptoms of pneumonia. HHF was one of the four international sites for this study and identified critical information about community beliefs and practices in rural Haiti. The results of this research were presented in 1991 to USAID, the Pan American Health Organization, the Haitian Pediatric Society and other NGOs, creating national interest in the conceptualization of pneumonia and use of cotrimoxazole (a simple, low cost and available antibiotic) for treatment of bacterial pneumonia.

Over the last 20 years, HHF has succeeded in demonstrating the feasibility of community-based treatment for pneumonia to the Ministry of Population and Public Health (MSPP). To demonstrate impact, the Centers for Disease Control (CDC) conducted a program evaluation in 1997 which found a 50% reduction in pneumonia specific death rates in the HHF program area of Jérémie, Haiti. The CDC also used the HHF pneumonia database to conduct clinical examinations of children with recurrent episodes of bacterial pneumonia [34]. This research identified wheezing as a major contributor to recurrent episodes of pneumonia, which led to evidence for the use of traditional home treatments for wheezing, such as reducing smoke exposure and using caffeine-based home remedies. In addition to the CDC documented reduction in pneumonia deaths, HHF demonstrated that community health workers (CHWs) could effectively diagnose, treat, follow-up and refer pediatric pneumonia cases [35]. In partnership with Management Sciences for Health, HHF brought evidence to the MSPP and advocated for approval of a community-based pneumonia treatment protocol for CHWs, which led to its inclusion in the national Integrated Management of Childhood Illnesses protocols in 2005.

HHF also used their research on the treatment of childhood pneumonia to inform the policies and procedures of bilateral aid organizations. In 2005, HHF was selected and funded by USAID to be the field training site for Haiti for community-based treatment of pneumonia. Physicians, nurse managers and CHWs from six Haitian NGOs participated in a week-long training where they learned about evidence-based clinical protocols, community mobilization activities, and program monitoring. Between 2012 and 2013, five more NGOs were trained at HHF on community-based treatment of pneumonia. The HHF experience has been cited in research and program documents in many parts of the world.

## Thought leadership

### Helen Keller International (Nepal)

Helen Keller International’s (HKI) Action Against Malnutrition through Agriculture (AAMA) project started in 2008 with funding from USAID’s Child Survival and Health Grants Program (CSHGP). The project targeted three districts in the Far West Region of Nepal and used an integrated, nutrition-friendly agricultural strategy to increase food security, dietary diversity and nutritional status through promotion of improved techniques for homestead gardens and poultry production together with nutrition behavior change [36]. HKI and its local NGO partners selected volunteer women to serve as village model farmers (VMFs) and trained them in homestead gardening and nutrition practices. The model farms were used as community-oriented learning centers for both agricultural training and discussions of healthy nutrition behaviors, in particular the importance of using food and income from homestead food production to improve the diet of family members. Government service providers, including agriculture extension workers and Female Community Health Volunteers (FCHV), were also engaged in training and behavior change communication for both agriculture and nutrition practices.

In 2010, HKI was granted funds from the USAID Mission in Nepal to add a governance component to the AAMA project aimed at strengthening national and local government understanding of the importance of food security and nutrition to health and development and participation in shaping supportive policies and programs. The project served as a concrete example of how cross-cutting strategies could be implemented at district and village levels, and its visible achievements informed the National Planning Commission’s roll-out of a national multi-sectoral nutrition plan. To coordinate nutrition and food security planning across sectors, joint planning structures were established at the regional and district levels, and these served as platforms for discussing department contributions to advancing and sustaining AAMA’s activities and for replicating or adapting the model for other areas.

Each of the government agencies working at the village level were invited to join a Nutrition and Food Security Working Group. This diverse group included the heads of the agriculture and livestock service center, the health facility, the district education office, representatives of HKI, political parties, FCHVs, VMFs, and many others. Together they reviewed the local nutrition problems, presented evidence for the AAMA model as a viable solution, discussed innovations emanating from the local communities, and reviewed government guidelines for budget allocation. As a result, government representatives at the district and village levels were better able to develop, monitor and supervise nutrition and food security development plans.

These working groups have been successful in promoting citizen participation in deciding budget allocations, and in bringing together leaders from multiple sectors to plan, coordinate, and influence VDC- and district-level funding. The Government of Nepal has continued to expand its multi-sectoral plan for addressing nutrition through agriculture and there is great interest on the part of international donors in supporting the replication of the AAMA strategy.

### Future Generations (Peru)

In 1993, the Peruvian Minister of Health saw the need and opportunity to introduce an alternative way to organize primary health care (PHC) services involving community participation [37]. For this, Future Generations assisted the MOH to apply a theory of community change known as “SEED-SCALE” which suggests a program design that: (1) builds on previous community success, (2) develops an effective local system based on community-government partnership, and (3) promotes local assessment and planning with task allocation. The local system is then used to scale-up with sustained impact [38]. The concepts of SEED-SCALE led to a new management model for government PHC services involving “Local Health Administration Communities” (Comunidad Local de Administración de Salud, or CLAS), which are structured as private, non-profit civil associations composed of elected members from both communities and local government entities. CLAS associations receive public funds to operate health services with legal accountability and have the capacity to hire and fire health staff, purchase medicines and supplies, and contract for improved infrastructure. The improved quality of care achieved through the CLAS model leads to greater demand for services.

Due to high satisfaction of clients and providers, CLAS rapidly scaled up to 250 by the end of 1994, 432 CLAS by 1997, and 743 CLAS associations administering 2153 PHC facilities (one-third of the total) nationally by 2002. To ensure the legal stability of CLAS, Future Generations began legislative advocacy in 2003 with the Peruvian Congress. In 2007, a new law formalized CLAS as national policy for co-management and citizen participation in PHC services [39].

In parallel, Future Generations built on the success of the CLAS model to improve impact on MNCH outcomes through community-based health promotion. An initial project in one CLAS-run health facility in Huánuco, Peru served as a pilot “Self-help Center for Action Learning and Experimentation” (SCALE), first developing a fully functioning and effective local system with local ownership (SCALE-One), then moving to the next level as an observational training center to extend knowledge and skills for scaling up (SCALE-Squared). This effort was later supported by two consecutive USAID CSHGP grants [40], which allowed for further innovations to the model and five training centers in other sites. Future Generations worked with MOH partners on criteria to certify PHC facilities as SCALE-Squared Centers for community promotion of MNCH. The Huánuco regional government issued an ordinance designating one of these PHC facilities, Acomayo, as a “Center for Development of Competencies in Health Promotion,” for observational training to scale the model to other PHC facilities in Huánuco. The model has won 11 national honors and awards for quality since 2005.

Future Generations continues to advocate for CLAS, leading a group of concerned NGOs, experts, and MOH staff to participate through an “Interest Group on CLAS,” and continues to develop this model of community health promotion with local co-management that is contributing to MNCH policy and practice in Peru.

## Joint venturing

### Concern Worldwide, International Rescue Committee, and World Relief (Rwanda)

In 2001, Concern Worldwide, the International Rescue Committee, and World Relief were implementing three separate projects funded by USAID’s CSHGP in three different districts of Rwanda, each focusing on multiple child survival interventions. In 2003, headquarters staff in the U.S. and field staff in Rwanda from all three organizations started discussing how these organizations could collaborate to achieve greater impact than each organization could by itself. A year later they were awarded a small pilot grant to test home-based management of malaria (HMM) by CHWs; the funding was from the CORE Group, a collaborative network of NGOs seeking to improve maternal and child health by sharing knowledge and building partnerships [41].

An evaluation of the HMM pilot project led to the recommendation that the approach be scaled-up to all 19 malaria endemic districts, and the Rwandan MOH subsequently received funding from the Global Fund to scale-up HMM. Throughout the pilot project, the three NGOs collaborated closely through cross-visits, collaborative monitoring and evaluation plans, and joint trainings. In 2006, the NGOs formed a consortium and submitted a joint proposal to the CSHGP, which resulted in a five-year initiative covering the three original districts (greatly expanded from the original projects, due to re-districting) plus three additional, neighboring districts, combined representing nearly 20% of the country’s total population. The new project, called *Kabeho Mwana*, built on the malaria intervention by adding pneumonia and diarrhea treatment by CHWs. In 2008, integrated community case management (iCCM) [42] for all three diseases was rolled-out in all six districts, and was scaled nationally by 2010 through the efforts of other national and international organizations and the Rwandan government.

The *Kabeho Mwana* consortium generated evidence from initial efforts to implement CCM for each of the three conditions, which served to inform broader policy uptake. While *Kabeho Mwana* participated in influencing institutional norms, the project also responded to changing norms by continuously adapting to evolving MOH policies around iCCM. With every adaptation to iCCM policy by the MOH, additional training and new CHW tools and registers were required. *Kabeho Mwana* continued to advocate and build evidence for iCCM to be considered as part of a broader community health system, including attention to nutrition, health promotion and CHW supervision [29].

*Kabeho Mwana* was successful due to the favorable political environment for community health and the consortium’s efforts to harmonize human resource policies. First, the Rwandan government prioritized community health, staffed its MOH hierarchy with dynamic and skilled administrators, and was committed to generating results. Second, the consortium of NGOs did not operate under the typical sub-award structure, rather they created a single human resource management system with a shared budget so that decisions could be made more easily and with transparency. Overall, this project was successful, both in terms of internal management within the consortium and in terms of support to and recognition by the MOH [43].

### ChildFund International, Plan International, Catholic Relief Services, Africare, Counterpart International and World Vision (Senegal)

Health huts in Senegal have been in existence since 1978, and were created to provide basic health promotion and selected curative services in areas without nearby public health facilities. However, MOH support for health huts was abandoned by the mid-1980s, and almost all (over 80%) were closed by the end of the decade. A new initiative to resuscitate the health huts was started in 1998 as a pilot project by ChildFund (then known as Christian Children’s Fund). ChildFund received funding from USAID’s CSHGP to support two projects (called Community Actions for Nutrition and Health, or CANAH I and II) which ran consecutively from 1998 through 2006 to work in three health districts.

In 2006, a large-scale partnership began with the Community Health Project (Programme Santé USAID/Santé Communautaire, or PSSC) in order to rapidly expand health services nationally and reach over three million people through health huts by 2011 (more than 25% of the national population). Funded by the USAID Senegal Mission, NGO implementation partners included World Vision, Africare, Plan International, Counterpart International, and Catholic Relief Services. The MOH was engaged at the national, regional and local levels, ensuring increased standardization of health interventions, the addition of more health interventions, and more complete integration of health huts within the overall national health strategy. By working in partnership, the consortium was able to take advantage of economies of scale by utilizing the resources and networks of each NGO in different regions of the country, thereby reducing costs and building on community trust. PSSC II continued through 2016 with many additional national and local partner agencies, and a goal of reaching over 70% of the national population, about nine million people.

The consortium of NGOs has further leveraged their work with health huts to ensure that vertical health priorities and resources are integrated into a community-based approach to improve national health at scale. Thus, as different priority health interventions have been funded over the years, such as child nutrition, malaria, and family planning, new activities have been added to increase health hut coverage. This level of partnership not only increases national coverage, but also helps coordinate the data collection and management of health services by standardizing training, implementation, staffing, monitoring, and reporting systems across multiple implementing partners.

In 2013, the MOH approved a national Community Health Policy, which established an overarching vision for community health in Senegal in the short and long term. The Community Health Policy recognizes health huts as an integral part of the national health system and formalizes the community health model, to which the NGO consortium has made significant contributions. The collaboration between six prominent NGOs not only increased health service coverage, but also increased their leverage in supporting national health priorities in Senegal.

## Drivers of institutional change

The six illustrative cases presented above provide examples of the three pathways through which NGOs and other actors can stimulate the institutionalization of community-focused MNCH strategies to strengthen health systems. In addition to these three pathways, there were also drivers of institutional change that were common across each case. Hartmann and Linn [11] define “drivers” as the factors that move a development intervention forward. The six drivers of institutional change described below focus on creating a cultural shift towards community-led accountability, establishing sustainable support systems, and fostering strategic alignment among development actors in order to move community-focused MNCH strategies forward.

### Strategic responsiveness

Each NGO was able to incorporate aspects of the other pathways into their strategic response by adapting to the national health priorities over time. HHF’s work in Haiti exemplifies how to combine “learning for leverage” with “thought leadership.” They successfully demonstrated the effectiveness of the community-based treatment of childhood pneumonia (i.e., learning for leverage) and, as a result, became a valuable source of technical training and information sharing from which other organizations could benefit (i.e., thought leadership). Concern Worldwide and its partners in Rwanda were able to strategically combine “joint venturing” with “learning for leverage.” The joint venture started with a group of like-minded NGOs that were implementing similar projects in Rwanda. Together they were awarded funds for a pilot program to test the effectiveness of HMM. After demonstrating its effectiveness, the NGO consortium had the opportunity to continue to expand other components of iCCM at the national level. In order to take full advantage of each pathway introduced in this paper, implementing organizations need to be responsive to the policy environment and make use of different pathways of institutionalization at strategic points in time.

### Partnership with policymakers and stakeholders

Political leaders need to be informed that it is in their interest to place community-focused MNCH strategies on their agendas [11]. The strategic involvement of key stakeholders was demonstrated by each NGO. For example, in Bangladesh, CARE invited senior level government officials, academic and research institution personnel, and other development partners to participate in workshops and visit project areas to better understand the success of CmSS. Through the AAMA project, HKI showed that bringing a diverse group of stakeholders from different sectors of the government can lead to an integrated, cohesive strategy to meet the nutritional needs of children in Nepal. It is important for implementing organizations to continue to look for opportunities to involve key stakeholders in all three pathways and look for “policy windows” through which they can leverage these relationships to integrate MNCH strategies into existing policy and practice. From the Ministry of Health’s perspective (as well as other Ministry officials), it can be beneficial to work in partnership with NGOs when strategic priorities align. This can lead to opportunities for government officials to receive on-the-job training on key aspects of community-focused MNCH strategies (e.g., community-based treatment of childhood pneumonia or home-based management of malaria) as well as opportunities for the expansion of MNCH strategies to geographic areas that are difficult to reach.

### Community involvement and ownership

Community-based development programs have become a growing priority for development assistance organizations, as demonstrated by the approximately 400 community-driven development projects supported by the World Bank in 94 countries valued at almost $30 billion in 2013 [44]. In order for investments in community-based development to be successful, “top-down resources and bottom-up capacity building need to be in a dynamic and cooperative relationship ([45], p. 185).” Two key components of community-based programs are community involvement and community ownership, which can increase the probability that local needs are appropriately reflected upon and taken into account [10]. In each case, community involvement and ownership led to community leadership, which is critical to the long-term success and sustainability of any community-focused MNCH strategy. For example, the Future Generations strategy involved the community from the beginning in leadership, planning, and monitoring of PHC services, by means of an initiative that linked community co-managed health services with home-based behavior change. Community ownership not only led to improvements in the quality and coverage of health services, but also contributed to the collective efficacy necessary to move the CLAS program forward when obstacles to implementation were encountered. In Nepal, HKI ensured that community members were placed in positions of leadership, such as village model farmers, to improve and diversify the diet of their family members and neighbors. Furthermore, HKI utilized VDCs to address food security and nutrition challenges in each village. As efforts to reduce maternal and child mortality expand into some of the most difficult contexts, community involvement and ownership will continue to be an essential component of the success of these programs. Ministries of Health should seek out partnerships with NGOs (especially local NGOs) to establish sustainable community linkages and identify community leaders with which to work.

### Monitoring and use of data

Special monitoring and documentation procedures are important to the process of institutionalizing new MNCH strategies [10, 11]. Monitoring can be used to ensure the intervention is being implemented as planned and to help adapt the intervention to the local context as needed. In addition, careful documentation of processes and results can help motivate communities, stakeholders, and other organizations to replicate the intervention. The CmSS approach used by CARE in Bangladesh is a perfect example of the importance of careful documentation of impact and purposeful dissemination. First, by tracking each pregnant woman in the initial phase of the project in Dinajpur, the community was able to develop a data system that monitored health service utilization. These data were not only used to help women in the community, but also served as a source of evidence to demonstrate the impact of the program. These data were then shared with donors and policymakers in order to demonstrate impact on a larger scale. Rigorous monitoring and evaluation of the institutionalization process itself is critical to the future success of community-focused MNCH strategies. Implementing organizations and governments should engage in systematic data collection of the long-term impacts of community-focused MNCH strategies as well as the process of institutionalization. Specifically, we encourage empirically-based research on the effectiveness of (or lack thereof) the three pathways described in our conceptual framework.

### Diversification of financial resources

In each case, the NGOs found multiple donors to fund each project, which contributed to success and sustainability. Two examples of the diversification of donors came from CARE in Bangladesh and ChildFund and its partners in Senegal, both of which were able to secure funding from a variety of donors to implement different phases of their respective programs. While financial resources are being diversified, it is also important for implementing organizations to have a consistent presence in each country so that additional resources can build upon what has already been established, rather than depending on funding streams that phase-out, stop, and start-up again [25]. Although it takes considerable time and money to develop and sustain relationships with multiple donors while maintaining a consistent in-country presence, donor diversification will continue to be important for public, private, and NGO partners to work together to strengthen health systems.

### Longevity

From Peru to Haiti to Senegal, these NGOs have worked in their respective countries for long periods of time, and many have been working on community-focused health system strengthening for over 20 years. Over time, they have been able to build relationships with government officials, communities, and other people of influence. Therefore, it is important to reconsider the current funding model of three to five year grants with the expectation for sustainable and scalable results. A new paradigm for funding needs to be established so that the expectation for long-term results is accompanied by a long-term financial commitment. This is especially important as many donors start to incorporate the idea of “scaling up” into their rhetoric for their strategic priorities [11].

## Conclusions

In March 2017, USAID and UNICEF in collaboration with the WHO, The Bill and Melinda Gates Foundation, and USAID’s flagship Maternal and Child Survival Program, hosted the Institutionalizing Community Health Conference in Johannesburg, South Africa. One of the primary objectives of this conference was to “advance the understanding of the opportunities and challenges for institutionalizing viable and resilient platforms for community health investments [46].” Our framework is a timely contribution to the on-going conversation about strengthening partnerships between governments and communities to ensure that every mother, newborn, and child not only survives, but thrives. The cases that we present demonstrate that NGOs and other implementing organizations can have an important role by complementing or partnering strategically with national governments to strengthen community-focused strategies, especially those that connect health systems and communities to address the SDGs and help accelerate the pace to end preventable maternal and child deaths.

In the health sector, it is not uncommon for NGOs to serve as direct implementers of community health programs in low- and middle-income countries that lack the capacity and infrastructure to adequately address preventable causes of maternal and child mortality [19, 20]. This role continues to be important in conflict-affected countries, and NGOs have demonstrated they can deliver services while supporting the national health system [47]. However, the model of using NGOs as direct service providers has at times been criticized for disrupting local health systems due to poor coordination and competition for resources [48]. This has led to a renewed call for NGOs to shift from solely direct service delivery to a model that leverages their strengths and experiences to have a sustainable impact on national government health systems where and when possible [9, 19].

We presented a conceptual framework that describes how NGOs and other implementing organizations can contribute to scaling up community-focused health service delivery, prevention and promotion strategies by institutionalizing these strategies to strengthen health systems at the district, national, or global level. Community-focused strategies are highly contextual and transaction intensive requiring a “learning by doing” culture as part of the scaling-up process, one that values adaptation, flexibility, and openness to change—attributes that many NGOs bring to the health and development process [11]. We provided six illustrative cases that demonstrated how NGOs contributed to institutionalizing MNCH initiatives through three non-exclusive, complementary pathways and six drivers of institutional change. In the first pathway, “learning for leverage,” NGOs demonstrated the program effectiveness of new innovations that influenced changes in the health system through adaptation of research into policy and practice. In the second pathway, “thought leadership,” NGOs disseminated lessons learned to other health and non-health partners through training, information sharing and collaborative learning. In the third pathway, “joint venturing,” NGOs worked in partnership with each other and the government health system to demonstrate the efficacy of a project and used their collective voice to help guide decision-makers. Regardless of the pathway used, six key drivers of institutional change were found to be critical for successful NGO scale-up: strategic responsiveness to national health priorities over time, partnership with policymakers and other stakeholders, community ownership and involvement, monitoring and use of data, diversification of financial resources, and longevity of efforts.

We propose that this framework can be used by donors, governments, and NGOs to encourage collaboration and contribute to program planning and policy making for the institutionalization of community-focused health strategies in the SDG era. However, additional empirical research on these three pathways and the six key drivers of institutional change is needed to provide an evidence-base for the process of institutionalization. Rigorous qualitative methods—such as case study research or phenomenological research—would allow us to start unpacking the complexity of each pathway and learn how each pathway has succeeded (or failed) to strengthen health systems.

## Abbreviations

AAMA: Action Against Malnutrition through Agriculture
CANAH: Community Actions for Nutrition And Health
CBHC: Community-Based Health Care
CDC: Centers for Disease Control
CHW: Community Health Workers
CLAS: Local Health Administration Communities
CmSS: Community Support System
CSHGP: Child Survival and Health Grant Program
FCHV: Female Community Health Volunteer
HHF: Haitian Health Foundation
HIV/AIDS: Human Immunodeficiency Virus/Acquired Immune Deficiency Syndrome
HKI: Helen Keller International
HMM: Home-based Management of Malaria
iCCM: Integrated Community Case Management
JICA: Japanese International Cooperation Agency
MNCH: Maternal, Newborn, and Child Health
MOH: Ministry of Health
MSPP: Ministry of Population and Public Health
NGO: Non-Governmental Organization
PHC: Primary Health Care
PSSC: Programme Santé USAID/Santé Communautaire
SDG: Sustainable Development Goals
UNICEF: United Nations Children’s Fund
USAID: United States Agency for International Development
VDC: Village Development Committee
VMF: Village Model Farmer
WHO: World Health Organization
